# Supplementary material for: Polyethylene Terephthalate Hydrolases in Human Gut Microbiota and Their Implications for Human Health
Source: Microorganisms. 2024 Jan 10;12(1):138. doi: 10.3390/microorganisms12010138 (PMC10820491; doi:10.3390/microorganisms12010138)
Supplement: Supplementary file 1 [file microorganisms-12-00138-s001.zip › microorganisms-2789005-supplementary.pdf]

Supplementary information

Polyethylene Terephthalate Hydrolases in Human Gut Microbiota and

Their Implications on Human Health

Table S1 Nucleotide sequence of HG1-5.

Table S2 Primers used in this study.

Figure S1 Neighbor-joining phylogenetic tree based on the amino acid sequences showing the phylogenetic location of 40 sequences.

Figure S2 Distribution of catalytic triplets and surrounding amino acids. Green represents catalytic triad S-H-D.

Figure S3 Multiple-sequence alignment of HG2-4. Red markings indicate conservative sequences.

Figure S4 Effects of terephthalic acid (TPA) and mono(2-hydroxyethyl) terephthalic acid (MHET) on viability of Caco2 cells and FHC cells.

Figure S5 Relative expression levels of IL-1β, IL-6 and TNF-α in RAW 264.7 cells with different treatments.

Table S1 Nucleotide sequence of HG1-5.

Sample number	Sequence
HG-1	ATGCAAAATCCTTCCGCCCCTGTGGTTGAAACGCGCCAGGGCGCA<br>CTGACTGGTTTAACCGATGAAAATGTCCACCGCTGGTGCGGTATTC<br>CCTATGCTGCACCGCCAGTGGGTGACTGGCGCTGGCGCTCACCGC<br>GCCCACCAGAACGCTGGGATGGCGTTCGCGATGCCACTGCCTTTTC<br>GGCCTCAAGCTGGCAAAGCAGTGAATATTGTCAGGAGCTGGGCGG<br>GGGCGATCCCGGCCAGTTCTCTGAAGACTGTCTCTATCTGAATGTC<br>TGGTCCCCCGTCGACCGCCCGGGATCGCTTCCGGTGATGGTCTGGC<br>TGCACGGGGGCGGGTTTACGATTGGTGCCGGTGGTCTGCCACCGT<br>ATCACGGCAAATCCCTGGCCGCGCGCGGCGTGGTTGTGGTCACGA<br>TTAACTACCGTCTCGGCCATCTCGGCTTTTTCGCCCATCCCGCGCTG<br>GAGGGGGAAGAAGAGCGGGTAGTGCATAATTTTGCTCTGCTTGAT<br>CAGATTGCCGCGCTTGAATGGGTGCGGGAGAATATCACCGCGTTTG<br>GGGGCGACCCGCATAACGTGACCCTGTTTGGTGAGTCCGCTGGCG<br>CCCGCAGCGTGCTGTCGTTGCTGGCCTCGCCGCTGGCGGAAGGGC<br>TGTTCCACAAGGCGATAGTCCAGAGTGGATATACCTTGCCCGACAC<br>GCCGCGCCAGCAGGCGCTGCAAAAAGGGGAAGCACTGGCGGCCC<br>ATTTTGGGCTGGAGAACGCGACGGCAGAGCAGCTGCGCGCGATCC<br>CGCCTGAGTCATTCTGGCCGCTGACCGCGCCACTCAACATCGCCCC<br>GGCGCCGATCGTCGGGGATTGCGTCCTGCCGGAGGCCATGCTGGA<br>TGTCTTTTTCGCTGCCCGCCAGCACCCTGTGCCGGTGATGATAGGT<br>TCTAACAGCGATGAAGCCAGCGTCATGGCGGTGTTTGGCATCGATC<br>TCGCCGGGCAAATCCAGAAACTTCGCCGGGAGCGCCGTTTTGGGC<br>TGGGGCTCATCAAGCTGCTTTATCCGGGTGTGAAAGGCGACGAAG<br>AGCTGGGCAGACAGGTGTGTCGCGATATGGCGTTTACCACCATGG<br>GCTATGTCGTGATGCAGGCGCAGCAGCGTGTCGGCGGGCTTTGCT<br>GGCGTTACTGGTTTGACTATGTCGCTGAAGCGGAGCATGCCACCTA<br>TATCAACGGCGCGTGGCATGGCAATGAAGTGCCCTACGTGTTCGAT<br>ACCCTCGGCCAGGTGGAGCCTTCACGTCAGTATGTCAACGAACGG<br>GATTTACAGTTTGCTGCCAGGGTAGCCGATTACTGGGTAAGCTTTG<br>CGCGCGATGCCGGAACGCATGATAGCCTGTCGGGGCCGACGCACT<br>GGCCCGCGTGCCGTAAAGGACGGGACGTGCTGCTACGTATCGGTG<br>TGAATAAACATGCAGGTTTCAGGCTTGAAAACCGCTTTATGCGCGC<br>GCGCATGAGCCTCTTTAAACGGGTGATGAAGCACCACGTCAGCCT<br>CGATTAA
HG-2	ATGCAGAAATACGTTGAAATCAAGCGCGACGGGTTAACCTTACGG<br>GGGATGCTGCACATTCCGAACGACGTTGTGAGCCAGAAAGTTCCG<br>ATGGTTATTCTGTTACACGGCTTTTGTGACGACCGCAATGAAATAA<br>ATTTTGTTCACAATGAGCTGAGTCAGAGACTGTGTGATGCAGGTAT<br>AGCAAGTGTTAGATTCGATATGAATGGTAGTGGTGAAAGCGACGGT<br>CGTTTTGAGGATATGACAGTTAGCAGCGAGATTTTAGACGCACAAG

	CAATGCTGAGATATGTTCGTAGCCTGGATTTTGTTGATACCAAAAA<br>GATTGCACTGCATGGTTGTAGCCTGGGTGGTTGTGTTGCATCAATG<br>GTTGCAGGTAAGTGTAAAGACCAGATTCGTGCCCTGTCCCTGTGGT<br>GTCCTGCGCCGGATCTGGTTTATAATCTGAAAGAACATAAGACCCT<br>GTGTAATCAGGATGTGAGTAATATTGAAAAGGATGGCTGCGCAGAT<br>GTTGAGGGTTTAAAACTTAGCCTGAAATTTTATCAGGACGCATGTA<br>CCCTGGATCCATATAAAGAAGCAAGCCTGTTTGATAAAAACGTTTG<br>CACGATACATGGTGATCAGGATATTACCGCAAGTTGCGAATGTAGC<br>TATAAATATAAAGAGATCTTCAAGGAGCGTGCAAAATGTATTATTGT<br>AAAAGGTGCAGAACACCGCTTTAAAAGCTTCGCATTTCGTGAAGC<br>ACGTATGCAGGGGGCACTGGATTTTCTGAAAGAAGAGCTGCTGTA<br>A
HG-3	ATGGAAAAGAGCGTGCTGATCAAAGGCGACAACTATGACATTAGC<br>GGGATTCTGAGCTATAAAGAAACCGTGGATAAAATGCCGGCGGTG<br>ATCCTGTGTCACGGCACCGGGGCACAGAAAAATGAAGTTGGTGAT<br>CTGTTTGTGATTCTGGCCGAAAAACTGCTGCAGCGTGGAATTGCA<br>AGCATTCGTATTGATTACGCAGGATGTGGCGATAGCAAGGCAGACC<br>AGCGGGAATTAACCTTTCTGGGTGAAGTTGAGGATACCAAAAAGG<br>CATACCAATATATATGTGATCTGGGTTGTGTTGATCAGAAAAATATT<br>GGAATTTTAGGGTTCAGCCAGGGAGCAAGAGTGGTTGCAGAATTA<br>CTGAAAGAGATGCAGGAATTTACCTGTGTTGCAAGCTGGAGTGGG<br>GCGTGTCAGAATGGTCGTGGCGTCTTCGAAGGGTGGTTTCAGGAA<br>TATTATCAGGAAGCAGAAGAACATGGTTATGCGAGAATCCCTATGG<br>GCTGGCGTGATGATCTGCTGCTGAGTAAGCAGTGGTTTGATGAAAT<br>TGAAAATACCACACCGATGGATGGACTGAAAAAGTATACCGGTCC<br>GGTACTGGCCGTTGCAGGTGCAGCGGATGAAATTGTGCCGTGTTC<br>CCATACCAAAGAAATCATGGCAGAAGGCACAAATGAACAAAGCAA<br>AATGCTGATCCTGCCGGGGGCAGATCATATTTTTAATGTGTTAAGCG<br>GTGATAAGACAATGAGTGAACATGTTTTAGATGTGACCGCAGACTG<br>GTTTGCAGAGGTGATGGGTGGAGCAAAAATTAGCAAATAA
HG-4	ATGGACGAAAACTACCCGGTTCTGCCGGGCGCGGACAGCTTTTTT<br>ATTAAAGGTAACGAAATCGGTATCCTGATTAGCCACGGCTTTAATG<br>GTACCCCGCAGAGCGTTCGTTTTCTGGGTAGAGCAATGGCAAGCG<br>ATGGTTTCACAGTTTGTGCACCGCGTCTGAAAGGTCATGGTACACA<br>CTACCTGGACATGGAACGTTGTACGTATAAAGATTGGATTGATAGC<br>CTGGAAGAAGGTTATCAGCTGCTGAAGCGTCATTGCCGTGATATTT<br>TTGTCATTGGTCAGAGCATGGGCGGTACCTTAGCGCTGCATTTAGC<br>CGAAAAACATCCGGATATTAGAGGAATGGTGTGTATAAATGCAGCC<br>ATTCAGTCAATTCCGGAACTGGAAAAATGTCTGGCAAAAGGCCGG<br>TATATTCAGGAGGGTCCACCGGATATTAAAGCAGCAGGTGTTCATG<br>AAATAGCATATGAGAAAGTGCCGGCAGCAAGCATACGGGAACTGC<br>TGAGCGTGATGAGAGAAACAAGAGAAAATTTAAGCGCAATTCACT<br>GTCCGGCACTGTTTTTTCAGAGTACCGAGGACCATGTTGTTCCGCC<br>GGAAAATACCGATTACATTGCAGCCCATATATTAGCGAGCCGTAAA

	AAGATAATCCCGTTACGTAACTCCTATCATGTTGCAACCATGGACC<br>ATGAAAAAGAATGGATTGCAGCACAATGTAGCAGCTTTGTGCAGG<br>AAATTGCCCATATTGAAAGCCGTACCGAAATTTAA
HG-5	ATGAAGAAGCTGCTGAAAGTGCTGCTGATTATTTTTTTAGTTATCGT<br>TGTGCTGGTGATTGGTCTGGTGATTTTTCTGACGATTGCGAGCGGG<br>AAGCAGAATGCACCGAAAGAGTATTGGAATGCAATTGCCAGCGAA<br>GGTACCATTGAGAAGGAGTATAATAAACTGGGTAGTTACGAATTTG<br>AGAGCAAAGTTTATGATGCACCAAAAGTGGACAGCCATGATAATA<br>ATTTTGTTGTGTATATGCCGAAGGAAGAAGGAACCTATCCGTTAGT<br>TGTTATGGTTAATGGTAGCGGTACGCCGTGGGATAAGTATAAAGCA<br>GTTTTTGAACATTTCGCAAGCTGGGGATATGTTGTTGTGGGGTGTA<br>ATTATGAAATAAGCTGGGATGGTAAGCATGCAAGTGAGACATTAGA<br>TTTTGCACTGAATACCAAAGAGATAGCAGATAAAGTTGATACCTCA<br>AAGGTGGCAGTTTGTGGTCATAGTCAGGGGGGGGAAGGGGCGTTT<br>AATGCAGCATTAGAATATGACAATAGCGATATGTATAAGGCAATAAT<br>AAGCCTGAGCCCGACGAATCAGGAGCTGGCGCTGGGGCTGAAATG<br>GGGATTTAATCTGGATACAGATGATATGTACGCATATAGACTGGAAA<br>ATGTTACAATTCCGACAATGATTATCGCAGGTACAGGTAAATTCGAT<br>AGCGAAACCGTTACACCCCTGTATAAGATGGAAGACATGTTTGAAC<br>AGCTGAATACCGATGTGGTTATGGCAAGACTGAGCAATAATGTTGA<br>TCATGGAGCAGTTCTGTACGAGGCAAATGGATATGTGATTGCATGG<br>CTGGACTATTATCTGAAGGGTATTGAAACCAATGGCACCGCATTTTT<br>TGGTAATGAAGCAGAAATCAAAAACAACACAAGATATCAGGATTT<br>CACCTCCCAGAAAGTTAAGTAA

Table S2 Primers used in this study.

Gene	Primer	Sequence
GAPDH	Forward	AGGTCGGTGTGAACGGATTTG
	Reverse	TGTAGACCATGTAGTTGAGGTCA
IL-1β	Forward	TCGCAGCAGCACATCAACAAGAG
	Reverse	TGCTCATGTCCTCATCCTGGAAGG
IL-6	Forward	CTCCCAACAGACCTGTCTATAC
	Reverse	CCATTGCACAACTCTTTTCTCA
TNF-α	Forward	ATGTCTCAGCCTCTTCTCATTC
	Reverse	GCTTGTCACTCGAATTTTGAGA
Arg-1	Forward	CATATCTGCCAAAGACATCGTG
	Reverse	GACATCAAAGCTCAGGTGAATC

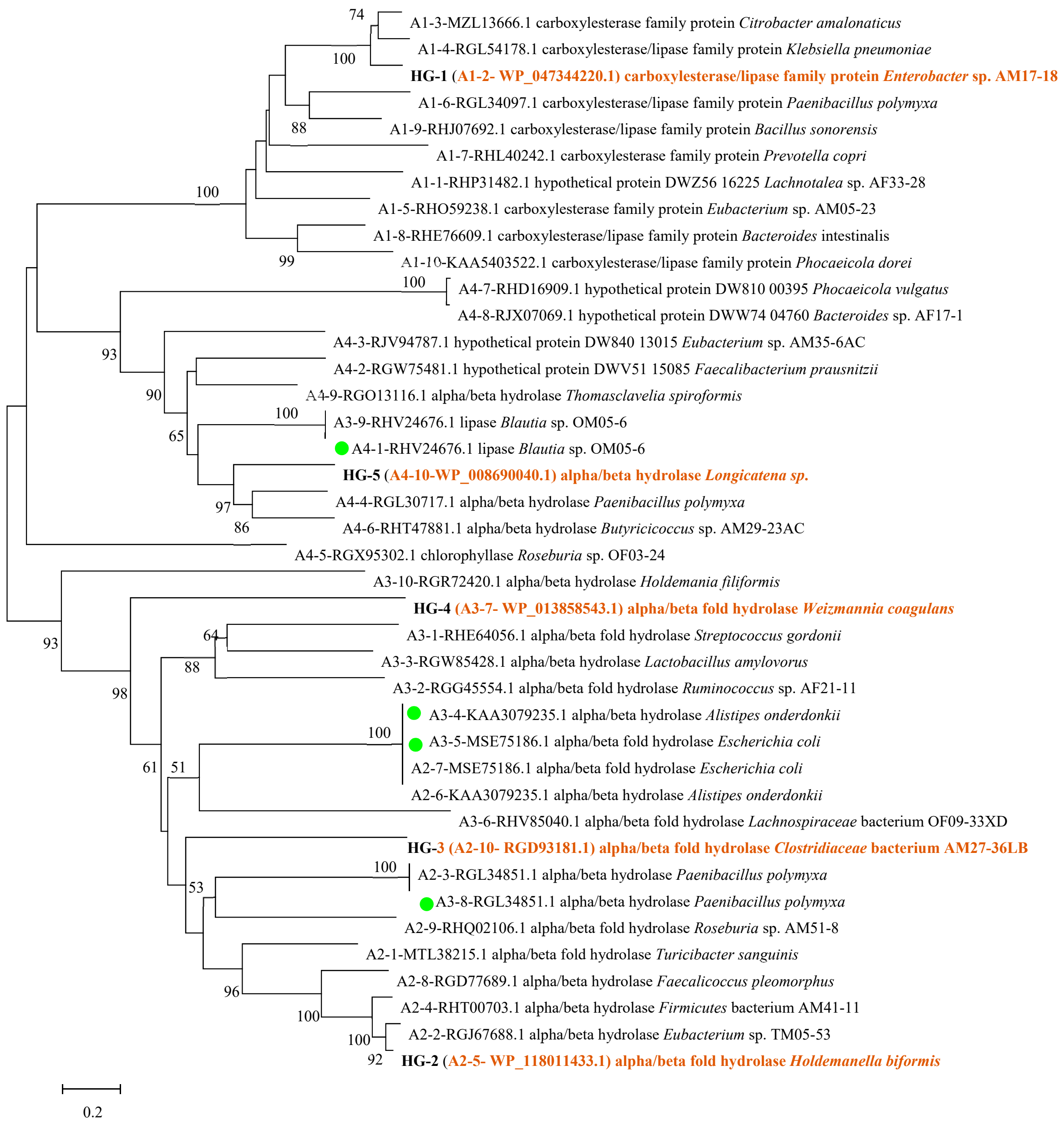

Figure S1 Neighbor-joining phylogenetic tree based on the amino acid sequences showing the phylogenetic location of 40 sequences. Numbers at nodes indicate the percentage of 1000 bootstrap replicates. Only bootstrap values above 50 % are shown. Bar represents 0.2 substitutions per nucleotide position. Green circles indicate repeating sequences. Pink bold font represents the sequence of expressed proteins.

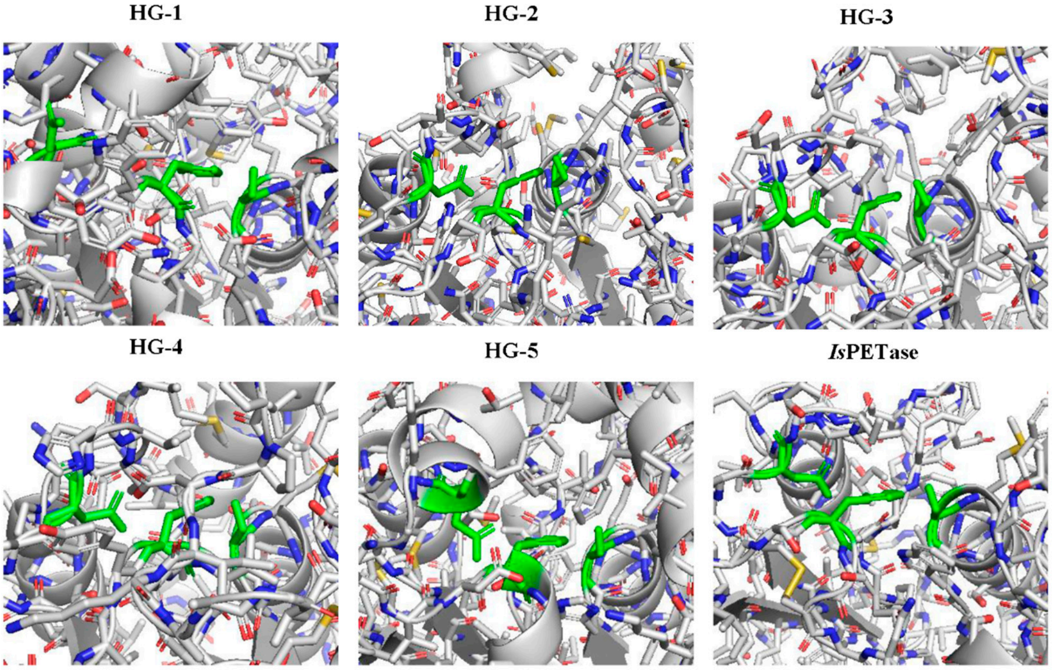

Figure S2 Distribution of catalytic triplets and surrounding amino acids. Green represents catalytic triad S-H-D.

```
         1        10        20        30        40        50
HG2   ..MQKYVEIKRDGLTLRGMLHIPNDVVSQKVPMVILLHGFCDDRNEINFVHNELSQRLCD
HG3   ..MEKSVLIKGDNYDISGILSYKETVD..KMPAVILCHGTGAQKNEVGDLFVILAEKLLQ
HG4   MDENYPVLPGADSFFIKGNEIG...........ILISHGFNGTPQSVRFLGRAMAS....

      60        70        80        90       100       110
HG2   AGIASVRFDMNGSGESDGRFEDMTVSSEILDAQAMLRYVRSLDFVDTKKIALHGCSLGGC
HG3   RGIASIRIDYAGCGDSKADQRELTFLGEVEDTKKAYQYICDLGCVDQKNIGILGFSQGAR
HG4   DGFTVCAPRLKGHGTHYLDMERCTYKDWIDSLEEGYQLLKRH....CRDIFVIGQSMGGT

     120       130       140       150       160       170
HG2   VASMVAGKCKDQ..IRALSLWCPAPDLVYNLKEHKTLCNQDVSNIEKDGCADVEGLKLSL
HG3   VVAELLKEMQEFTCVASWSGACQNGRGVFEGWFQEYYQEAEEHGYARIPMGWRDDLLLSK
HG4   LALHLAEKHPDIRGMVCINAAIQSIPELEKCLAKGRYIQEGPPDIKAAGVHEIAYEKVPA

       180         190       200       210         220       230
HG2   KFYQDACTLD..PYKEASLFDKNVCTIHGDQDITASCECS.YKYKEIFKERAKCIIVKGA
HG3   QWFDEIENTT..PMDGLKKYTGPVLAVAGAADEIVPCSHTKEIMAEGTNEQSKMLILPGA
HG4   ASIRELLSVMRETRENLSAIHCPALFFQSTEDHVVPPENTDYIAAHILASRKKIIPLRNS

         240       250
HG2   EHRFKSFAFREARMQGALDFLKEELL..........
HG3   DHIFNVLSGDKTMSEHVLDVTADWFAEVMGGAKISK
HG4   YHVATMDHEKEWIAAQCSSFVQEIAHIESRTEI...
```

Figure S3. Multiple-sequence alignment of HG2-4. Red markings indicate conservative sequences.

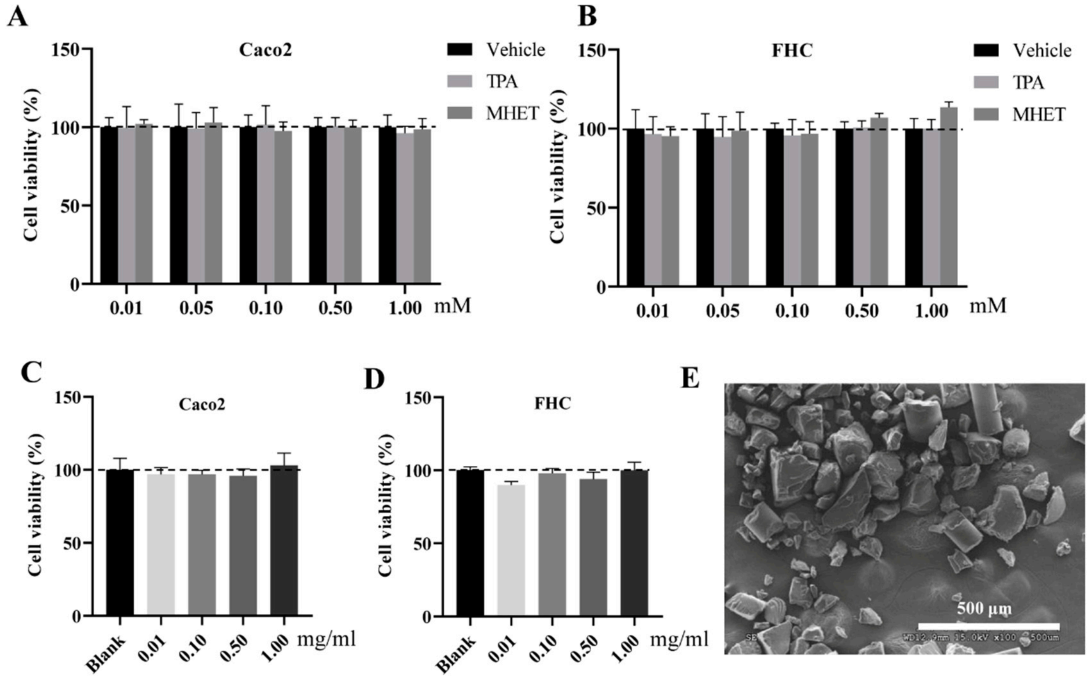

Figure S4 Effects of terephthalic acid (TPA) and mono(2-hydroxyethyl) terephthalic acid (MHET) on viability of Caco2 (**A**) and FHC cells (**B**). The same concentrations of DMSO (maximum 0.1%) were added to the medium for the vehicle control. Viability of (**C**)Caco2 cells and (**D**) FHC cells in response to PET MPs (< 400 µm). Mean values ± standard deviation of n=6 independent experiments were given. Statistical analysis was conducted by One-way ANOVA followed by Tukey's test. **E** SEM images of PET MPs particles.

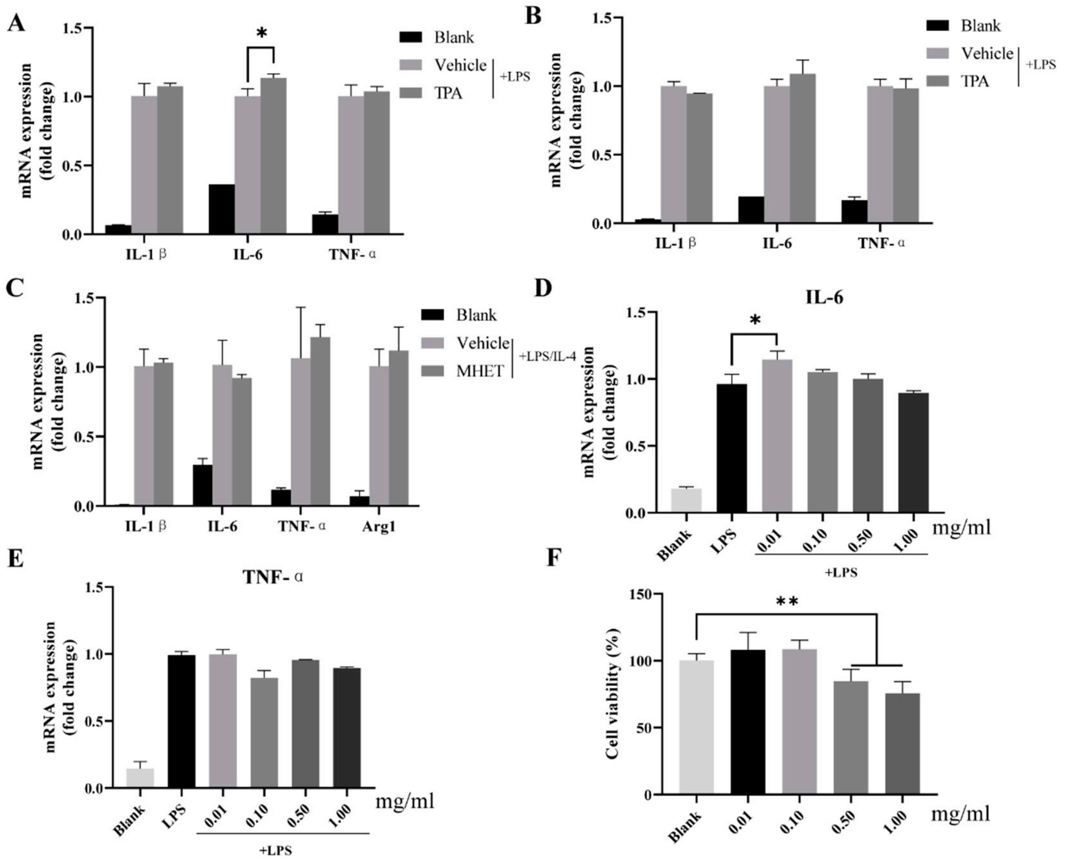

Figure S5 Relative expression levels of IL-1β, IL-6 and TNF-α in RAW 264.7 cells treated with TPA (0.10 mM) (**A**)and TPA (0.50 mM) (**B**)for 24 hours followed by lipopolysaccharides (LPS) stimulation for 6 hours. **C** Relative expression levels of IL-1β, IL-6, TNF-α and Arg-1 (IL-4 stimulation) in RAW 264.7 cells treated with MHET (1.00 mM) for 24 hours followed by LPS/IL-4 stimulation for 6 hours. Relative expression levels of IL-6 (**D**) and TNF-α (**E**) in RAW 264.7 cells treated with PET MPs (< 400 μm) at a gradient dose at 0.01, 0.10, 0.50 and 1.00 mg/ml for 24 hours followed by LPS stimulation for 6 hours. **F** Viability of RAW 264.7 cells in response to PET MPs (< 400 μm) at a gradient dose at 0.01, 0.10, 0.50 and 1.00 mg/ml. **p-value < 0.01; * p-value < 0.05;
